# Influence of Long-Term Storage on the Caking Properties Determined in Punch Test and Fungal Contamination of Potato Starch and Wheat Flour

**DOI:** 10.3390/ma14020331

**Published:** 2021-01-11

**Authors:** Justyna Wajs, Jacek Panek, Magdalena Frąc, Mateusz Stasiak

**Affiliations:** Institute of Agrophysics, Polish Academy of Sciences, Doświadczalna 4, 20-290 Lublin, Poland; j.wajs@ipan.lublin.pl (J.W.); j.panek@ipan.lublin.pl (J.P.); m.frac@ipan.lublin.pl (M.F.)

**Keywords:** punch test, powder materials, mechanical properties, fungal spoilage, powder quality

## Abstract

The presented results are an attempt to identify the changes taking place during a punch test experiment and the development of fungal impurities of powdered food materials over long-term storage at 75% RH. The potato starch and wheat flour market has a large share of the global production of bulk materials. The growing interest in powdered food materials requires additional production expenditure. This is associated with an increase in storage time of the discussed product and providing it with the appropriate conditions. The samples of potato starch and wheat flour were stored in perforated containers in a climatic chamber at 75% humidity and 21 °C for five months and then samples were measured by a punch test in a Lloyd LRX materials testing machine. The graphs obtained in the potato starch punch test differed significantly from wheat flour. The thickening of potato starch was observed in the form of layers, while potato starch was uniformly thickened throughout the experiment. The conditions of 75% humidity and 21 °C can be described as the beginning of the caking process. In potato starch, linear sections were observed, which changed the length of their storage time and, additionally, was correlated with the appearance of fungal contamination. These results may suggest the influence of fungi on the phenomenon of bulk material caking.

## 1. Introduction

Statistics show that the consumption of wheat flour in 2018 reached 385 million tons, while the potato starch world market reached 3.7 million tons. Both of these food powders play an important role not only in the food industry, but also in other industries such as pharmacy, the textile and paper processing as well as the chemical industry. Moreover flour and starch are increasingly being proposed as carriers for microorganisms in production of biopreparation for agricultural use [1,2]. The growing interest in potato starch and wheat flour requires additional production expenditure, which is associated with an increase in the storage of these products and providing them with appropriate conditions. Long-term and poor storage of the material may affect the quality of the products [3]. In the case of some raw materials, such as fruit, it may also involve changes in flavor parameters [4]. Therefore, it is extremely important to maintain optimal conditions and forms of controlling them by using modeling and simulation methods. This can contribute to a better understanding of the material behavior during the technological process [5]. Food powders are very sensitive to temperature and humidity. Too high humidity can result in adsorption of water by material particles and cause a decrease in the flowability of the material and, in particular cases, even cause caking of the material [6,7,8].

Additional problems associated with improper storage conditions that result in the deterioration of product quality are biological agents. One of them is microorganisms, especially molds, whose spores can be present at any stage of the production process. When temperature and humidity exceed to the minimum limit for the development of microorganisms, they start to grow. For mold, the lower limit of relative humidity can start from 60–70% [9]. Therefore, it is necessary to maintain proper storage conditions of the material throughout the entire processing process. For powders, it is assumed that the optimal warehouse temperature should be 15 °C and humidity about 55–65% [10]. Exceeding these limits can promote the development of mold and also cause condensation of material on the walls of storage vessels.

The most recent research described in the scientific literature is focused on the issues concerning the influence of humidity on physical parameters of powders and changes in their composition, as well as their influence on powder flowability and caking degree. Methods used to assess caking may include rheometric tests [11], blowing tester [12], compression and destruction tests [13,14], sample penetration tests [6,15], as well as other experimental methods and their modifications. In our research we used penetration test. The punch test method, which was originally proposed by Manahan, Argon, and Harling [16], was used to characterize metal elements in the power industry. Currently, this method is used and developed for different branches such as the evaluation of plastic properties [17] or in the evaluation of powder materials such as milk powder [15] or sucrose [12], as well as modifications of this method based on the same principle of operation are used for hardness and texture testing [18,19].

There are also studies on water activity and microbiological impurities in powders. Abdullah et al. [20] studied the effect of water activity on the degree of fungal contamination during storage on six starch-based food products. Sautour et al. [21] conducted studies on the effect of temperature and water activity of isolates from pastry products. However, there is a lack of experiments that combined the study of mechanical properties with the microbiological aspect of food powders. In our previous work [22], we tried to find a relationship between cake strength of agglomerates, structural changes, and fungal contamination during short-term storage in high humidity. This study is a continuation of mention topic, which was never examined according our best knowledge. For this purpose, we performed caking tests over a longer period of time and under lower humidity conditions, in order to mimic the standard conditions in the environment where these type of powders are stored. Penetration tests were carried out to determinate the degree of contamination tested materials with fungi and to try to link observed changes with the results of the penetration tests, which has not been undertaken in the literature so far.

In this study, we are focused on the relationship between the degree of caking of food powders correlated with the development of fungal impurities during long-term storage in the highest humidity limit accepted as safe in typical warehouses (RH 75%). The determination of the caking was examined by the punch test. The aim of this study was to evaluate the changes in potato starch and wheat flour during the punch test after storage them under controlled conditions. Additionally, related occurred changes to fungi contamination of the tested material, as well as to assess and identify the depth of caking in material samples. Powders, which are the most commonly used materials in the loose materials industry, have been selected for testing based on their different properties. Potato starch is a more homogeneous system, while wheat flour has other ingredients besides starch. In addition, these materials have a different morphological structure of molecules, so both of them, although they belong to powders, represent different properties.

## 2. Materials and Methods

Potato starch (PPZ, Trzemeszno, Poland) and wheat flour type 500 (Młyn Szczepanki, Łasin, Poland) were used in the study. The materials were dried in a SUP-30 laboratory dryer (Wamed, Warsaw, Poland) to a maximum moisture content of 3% ± 0.5. The process of drying the powders to achieve the aforementioned humidity level was aimed at unifying the water content of the samples, so that in each sample the initial water content was at the same low level. Humidity measurements were performed using Mettler Toledo HG63 moisture analyzer (Laboratory and Weighing Technologies, Greifensee, Switzerland). Cylindrical perforated vessels with a diameter of 24 mm, a height of 30 mm and mesh diameter 1.5 mm were used in the study, the purpose of which was to maximize the exposition of the material to humidity. The perforated cylinders had previously been sterilized in an autoclave (FVG2, Fedegari Autoklaven AG, Switzerland) to eliminate microorganisms originating from the vessel. The prepared materials were sieved through a sieve into measuring vessels to create more homogeneous deposit. Each vessel was completely filled with powder, whose net weight was about 6 g. Next, prepared samples were stored in a climatic chamber (HCP240, Memmert, Germany) at 75% humidity and 21 °C for five months. These conditions were intended to resemble the upper limit of the conditions prevailing in warehouses [9,10]. The measurements in the first phase (first 30 days) of the experiment were performed every two days, and then once a week. On each of the selected days, the measurements were taken in three repetitions. The control samples for both materials were dried to a maximum of 3% humidity and not stored. They were marked as day 0.

**Figure 1:**
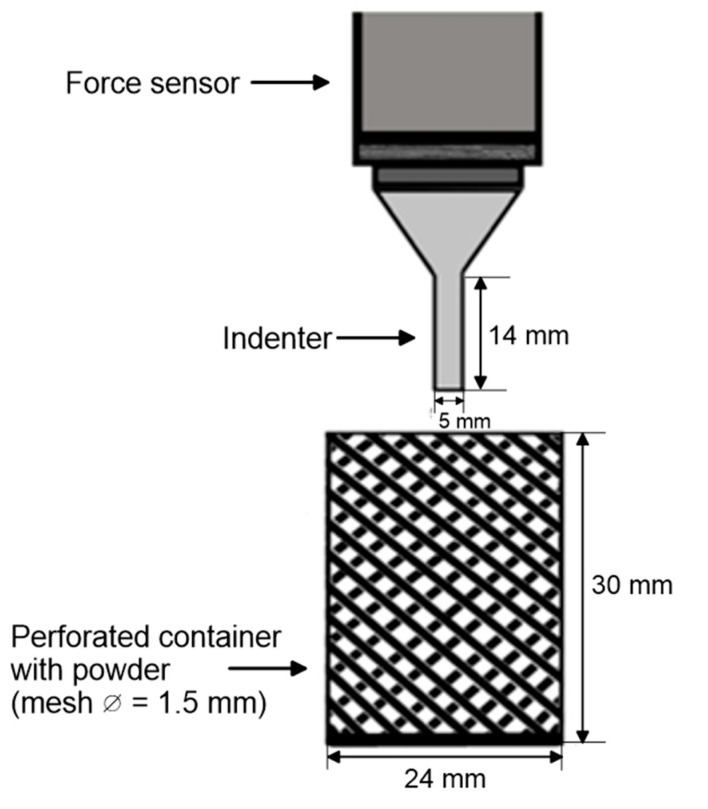
The measurement scheme of the punch test [22].

The procedure of measurement by means of a punch test is based on the method presented by Wajs et al. (2020) (Figure 1). The samples of material in perforated containers after prescribed storage time were tested in Lloyd LRX Materials Testing Machine and using Lloyd Nexygen software (Lloyd Instruments Ltd, Hampshire, UK). In the experiment, a 50 N load cell was used with a cylindrical shape indenter connected (diameter 5 mm and length 14 mm). The indenter moved into the material at a constant speed of 30 mm min^–1^. The measurement started when the indenter touched the surface of the material in the vessel and was carried out until a depth of 14 mm.

Measurement of the degree of fungal contamination was assessed based on the determination of the number of yeasts and molds by counting colonies according to the protocol described in the ISO 21527:2009 [23]. Samples after storage for prescribed days were collected, homogenized (Biocorp, Warsaw, Poland) and then diluted 10-fold in distilled water. A serial of dilutions were made and 100 µL of suspension was plated on sterile potato dextrose agar (PDA) medium (BioMaxima, Lublin, Poland) prepared in a Petri dish. The spread-plate technique with an L-shaped plastic, sterile pad was used to spread dilutions on the PDA medium. The prepared material was incubated for seven days at 25 °C. A 10^−1^ dilution was chosen for the analysis, from which the colonies were counted after five days as recommended by the standard. The measurements were made in triplicate. After counting, the grown colonies were converted to colony forming units (CFU) per gram of dry matter (CFU g^−1^) of tested material.

The obtained results were calculated with averages together with standard deviation, and then analysis of variance was carried out using software Statistica (v.13, StatSoft, Cracow, Poland). Information on the statistical significance was given as 0.95 confidence intervals shown as dotted lines on the graphs. *p*-value is the calculated probability (or level of statistical significance; *p* < 0.05 means statistically significant). A moving average was used to assess the development of fungal contamination. The use of a moving average was to mitigate the impact of random fluctuations in the results. The obtained CFU results were analyzed every six periods. Additionally, a matching line was inserted with the 95% confidence intervals (dotted lines).

## 3. Results and Discussion

**Figure 2:**
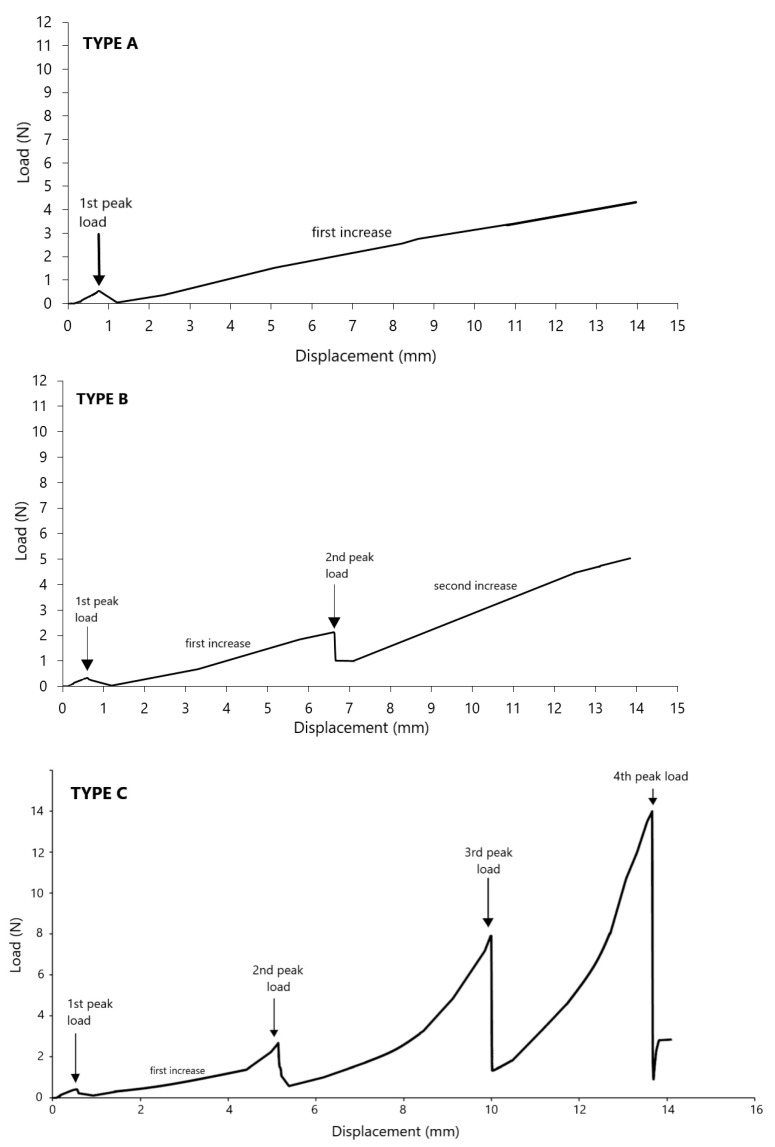
Model experimental runs of the punch test of the potato starch.

In the first stage, the runs of the punch test were analyzed to estimate changes of the strength value that characterized the studied materials. Among the obtained graphs representing the discussed features of both powders, we could distinguish groups characterized by similarity. Three types of graphs describing potato starch were obtained. The model groups of the graphs presented in Figure 2 represent typical graphs obtained in the experiment.

**Figure 3:**
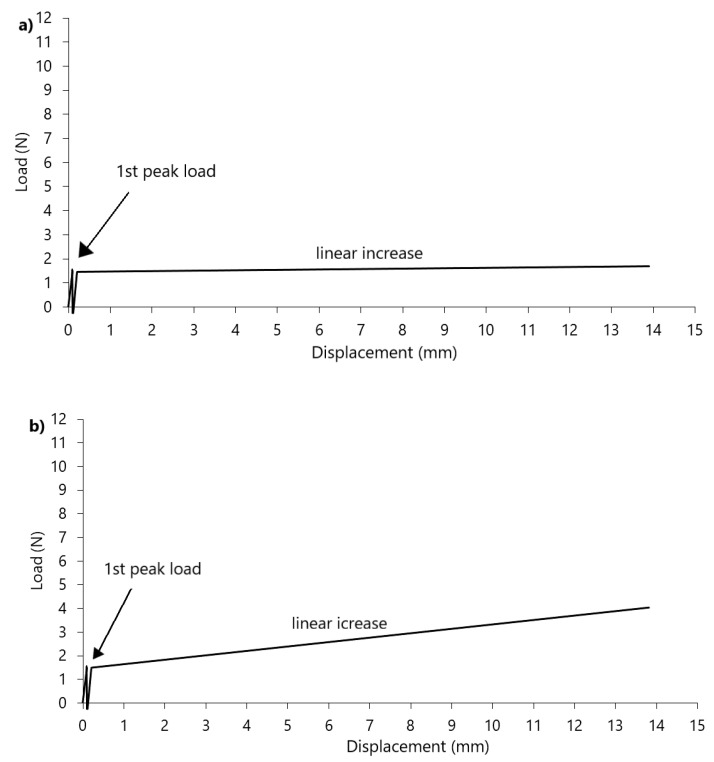
Model experimental runs of the punch test of the wheat flour: (**a**) control sample, (**b**) graph of other measurements.

The potato starch graphs are characterized by specific points that can be observed in each of them. Peak loads are the maximum values of periodic increases in strength, which are related to the gradual compaction of the material. The diagrams also show the first increase and the second increase, which describe the approximately linear sections of the increase between the peaks. Type A was characterized by one peak and one linear increase. This type of graph can be observed from days 1–7. From day 9, type B diagrams have been selected. They appeared until day 84 and were characterized by the presence of two peaks and two linear increases in force values. For the period over 84 days, type C graphs appeared. At the beginning, they were characterized by three peaks, while from day 114 of storage, another additional peak started to appear. Additionally, during the whole experiment in potato starch, a linear increase was observed after the first peak. The second growth developed over time and resembled an exponential function. The wheat flour treatments are markedly different from those observed for potato starch (Figure 3). Different model diagrams for time intervals cannot be distinguished here. The control sample (material dried to 3%, not stored) is slightly different from the others. In each run, one peak could be observed immediately after the start of the measurement. This is the moment when the sensor breaks through the powder surface. Then the force decreases sharply. In the control sample, the further part of the graph runs almost parallel to the x-axis without any further increase in force, while in the following days of storage the increase is also linear, however, increases in force can be observed. Only in some cases, in the final phases of the graph, disturbances were visible, but they decreased during storage time.

**Figure 4:**
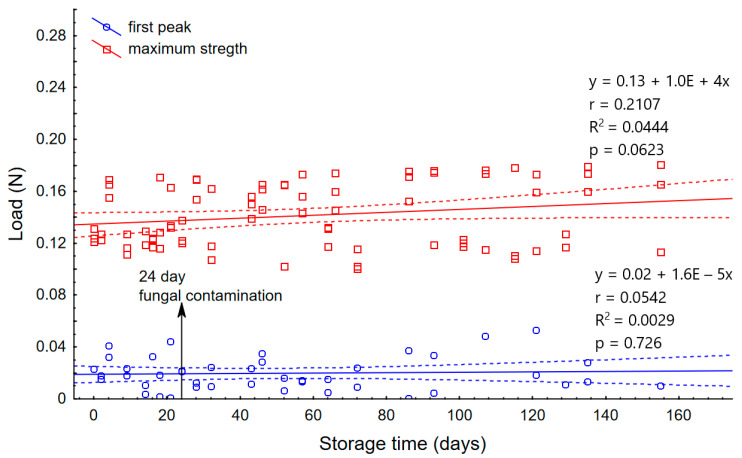
Average force values for the first peak and maximum force values for wheat flour. The dotted lines represent the 0.95 confidence intervals.

Along with the time of storage in elevated humidity, the potato starch is compacting and strengthening, resulting in an increasing number of peaks. The first peak load appears throughout the experiment and takes very low values up to 1 N. It was observed at a similar depth not exceeding 1.5 mm. It is, therefore, bound to the surface of the powder, and its values suggest the moment of penetration through the potato starch surface. The penetration tests of lumped skimmed milk powder [15] also showed an increase in strength at a depth of up to 1 mm, followed by a secondary increase. This suggests that also in this case we are dealing with a surface puncture of the powder. The strength graphs for wheat flour differed significantly from potato starch. During wheat flour strength measurements, no increased number of peaks was observed. This means that the caking in this material is uniform throughout the sample volume, while in potato starch the clumping is in layers. The punch test measurements in wheat flour showed single peaks at the beginning of the measurement. This sudden jump in the value is the moment when the sensor breaks through the powder surface. Statistical analysis of peak strength values between individual days did not show significant differences. The maximum peaks from wheat flour did not exceed 0.06 N (Figure 4). These low values of surface strength of the powder layer are characteristic for these type of materials, while the strength of other materials such as fruit and vegetable tissues [24] show much higher values. Additionally, the first initial peak can be observed in plastic or metal punch tests [17,25], where the initial values differ significantly from those of powders. The maximum strength values for wheat flour (Figure 4) were obtained in the final phases of the graphs at the end of the measurements and at the same time they were the end of linear increases. The obtained maximum values ranged from 0.1 to 0.2 N and showed weak (r = 0.2107) linear correlation with storage time. Similar strength values for wheat flour were obtained by Fitzpatrick et al. [26]. In their studies, wheat flour was exposed to 76% RH. The measurement was done at a rod diameter of 5 mm, which was moved downwards with a constant speed of 30 mm/min through the thin layer of material (7 mm). After six days, the force measurement showed a value of 0.17 N, which is very similar to the result obtained in our experiment. Therefore, after storing wheat flour in the 75% humidity we can expect strength values in the range of 0.1 to 0.2N. Such a low strength value may be the result of the physical properties of this material. Wheat flour belongs to "non-sticky" powders and the moisture content provided in both experiments is not sufficient to form cohesive bridges between its molecules to increase strength [26]. Similarly, in the experiment with 100% air moisture content of wheat flour, there was a continuous increase of strength over the period of time and significantly lower values than in potato starch. This result may be associated with insufficient possibility to form bonds between the particles and weaker tendency to caking [22].

From the ninth day of storage, a second peak was observed in measurements of potato starch. Until the 78th day, it appeared irregularly at 4–13 mm depths, after the 78th day the peak depth decreased and it appeared more regularly at 5–7 mm. During the whole experiment, it took values from 1 N to 5 N. In addition, in the first and second peak, we can observe an increase in strength up to 18 days, which may be associated with an increase in material density. No further significant changes in these peaks may suggest that after this time a stabilized material layer was formed. After day 84, thickening of potato starch and the formation of the next layers were observed, which can be plotted as the third peak and from day 114 onwards as the fourth peak. The third peak load is the only peak that showed a downward trend in force values. This decrease may be related to the formation of the next layer of material (the fourth peak). In the case of peaks 1 and 2, the values increased slightly, until the moment of stabilization of the material’s core and the appearance of three peaks. When the third layer of material forms, the situation is similar. In the initial days of its appearance, the values will be at a similar level, but over time they do not stabilize and their value decreases due to giving way to the next peak and moving closer to the surface. In addition to the above-mentioned downward trend of mean values of the third peak, among the remaining peaks no statistically significant differences in the matching lines were observed and no correlation between the variables was shown. The resulting layers of material and increasing force values suggest that under the influence of storage with increased humidity, the material became more stable and gained strength. The layering and the thickening of potato starch can be the beginning of the caking process. This means that conditions of 75% humidity and 21 °C can be the beginning of the caking process. A similar conclusion was drawn by Billings et al. [12]. They tested samples of sucrose using punch test method. After three days in an environment with water activity above 0.75, an increase in strength occurred. The samples tested by them showed higher strength with an increase in humidity. This study showed that in sucrose samples caking can be initiated already in conditions with water activity above 0.75, but only above 0.8 does it become significant. A similar conclusion was drawn in our previous studies [22]. In high humidity higher strength values were observed in both wheat flour and potato starch. Additionally, increases in strength occurred much earlier than in the results presented in this study, which indicates the occurrence of caking. In our results, the presence of a few peaks in potato starch may also suggest the start of the caking phenomenon, however, similarly to the Billings et al. [12] study, above these values they begin to be significant.

A similar comparison of conditions of 100% RH and 76% at 20 °C was carried out by Fitzpatrick et al. [14] on skim milk powder. Additionally, in these studies, the difference in cake strength values was observed due to exposure of the material on different humidity. As a result of the penetration test and force measurement performed by the authors, different maximum cake strength values for 76% RH and 100% RH (6 N and 10 N, respectively) can be observed. The cake strength values, in this case, are almost twice as high. Comparing the maximum cake strength values of potato starch obtained in this study (about 16 N) in conditions of 75% RH and the strength value obtained in the study Wajs et al. [22] in conditions of 100% RH (about 30N), we can conclude that the strength, in both cases, almost doubled. Potato starch and skimmed milk powder belong to the so-called "sticky" powders, so we can assume that the powders belonging to this group will clump similarly, with cake strength values almost doubling concerning storage at 75% and 100% RH. This should be confirmed by additional studies.

**Figure 5:**
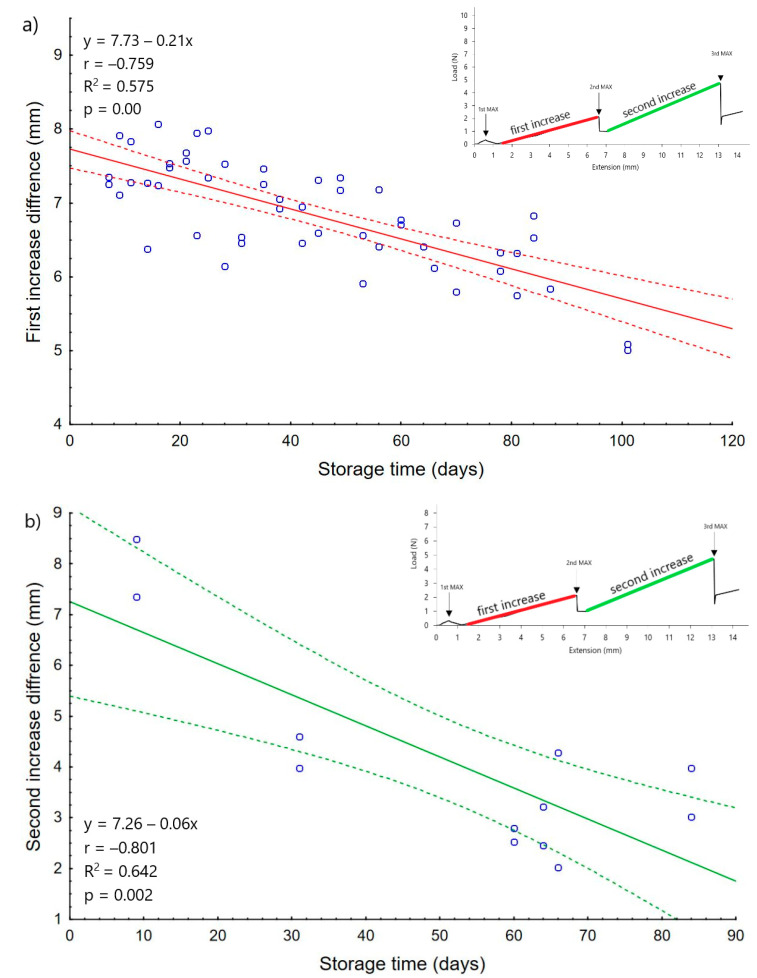
The average length of sections of the first (**a**) and second linear increase (**b**) of potato starch. The continuous line is the trend line, while the dotted lines are the 0.95 confidence interval.

**Figure 6:**
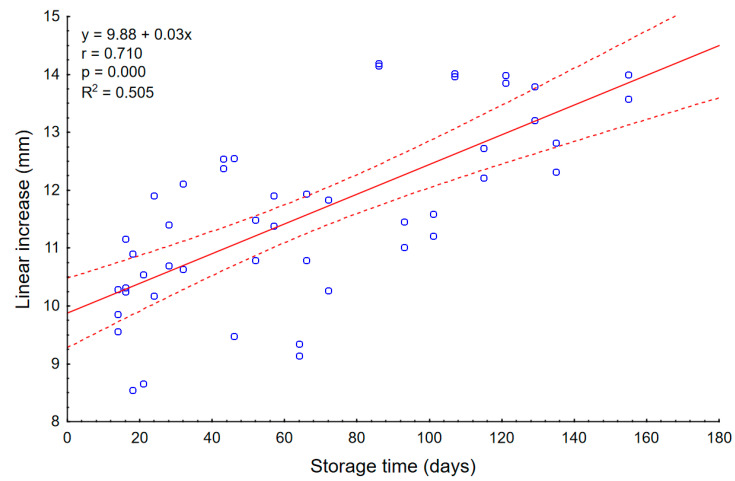
The average length of linear increase of wheat flour. The continuous line is the trend line, while the dotted lines are the 0.95 confidence interval.

An additional element that was observed in potato starch during the punch test were linear increases in strength. The analysis was based on the differences between the end of the growth and the start point. The calculated results are shown in Figure 5 and Figure 6.

Both the first and second increase decreased during the time of storage of the tested potato starch. A linear matching function is also inserted into the chart, which also indicates that this distance is shortening. The shortening of the linear sections suggests that the material strengthening has become more homogenous.

Figure 5a shows the values obtained for the first linear increase that took place until day 114 and appeared after the first peak load. In the first days of the experiment, this section was about 8 mm long, while with the storage time, a shortening was observed. Statistical analysis showed high (r = −0.759) negative linear and significant (*p* = 0.00) correlation with the storage time. This correlation may result from the appearance of the next peaks in the following days as well as the second linear growth, which results in the shortening of the first linear segment. The situation is similar for the second linear growth. Additionally, here we can observed a strong (r = −0.801) negative linear and a significant (*p* = 0.002) correlation with the time of storage. However, this increase did not systematically appear during the measurements. This decrease can also be caused by the formation of the next layer of material, which is illustrated in the graphs as the fourth peak load. Another explanation for the shortening of the second increase may be the fact that fungal contamination appears at the same time as this decrease.

An increase in the strength of the second peak has been observed since the date of appearance of the fungus. It is known that moisture can affecting the density of the material. It may suggest that the development of microorganisms could also affect the density of the potato starch layer and, thus, increase the strength value. A similar effect was obtained in the work Wajs et al. [22], where the amount of fungal contamination affected changes in the strength of the resulting agglomerates. The shortening of the first linear growth is, therefore, the result of the appearance of a dense layer, observed as the second peak. The relationship between the amount of fungal contamination, the second strength peak, and the shortening of the linear growth in potato starch is particularly well visible in the period up to day 21 where there is a temporary decrease in both the strength of the layer and the number of fungi in materials. In the case of wheat flour, this analysis was also carried out (Figure 6). As in potato starch, the end and beginning of the linear section were taken into account. Also in this case, a significant (*p* = 0.000) and a high (r = 0.710) linear correlation was observed between the linear section and storage time. The matching function indicates that the values of the linear growth section increased with the storage time and this is reflected in the graphs, as there were more disorders in the initial days of measurement in the end of the graphs. As the storage time passed, the test line was more "smooth", which may be related to the settling of wheat flour and its stabilization.

The wheat flour charts are mainly characterized by linear growth. Only in the initial phase of measurement, a force peak was observed, which was related to the sensor breaking through the powder surface. However, it can be observed that the linear section in wheat flour was increasing, which differs from potato starch samples values. In the first days of measurements, where the material was not yet sufficiently thickened, the sensor pierced the powder surface rapidly. This resulting in a long time for the indenter to adapt to the material and, thus, the length of linear growth was shorter. With the storage time, the material has consolidated and less susceptible to the sensor hitting the surface. Teunou et al. [7] studied consolidated powders including wheat flour in different humidity. In the results obtained, they classified the flour as a cohesive difficult flow powder, whose flowability does not change significantly in 25–66% RH. It can be assumed that the 75% humidity used in the above-mentioned experiment also does not significantly affect wheat flour and it still behaves like a cohesive difficult flow powder. The absence of peaks and only one linear growth in wheat flour can correspond to the model created by Kang et al. [27]. According to their tests and the model elaborated, all force-depth curves can be translated into a pressure-depth standard curve, resulting from Archimedes’ law. The results of their study suggest that the penetration of granular media will result in a non-linear initial phase and a later linear phase. In the presented experiment both wheat flour and potato starch represent non-linear phases and linear increases. According to Kang et al. [27], the differences in the transition to linear growths depend on the angle of internal friction of the powders tested and are a specific property of the bulk medium. Therefore, the curves obtained in this experiment may correspond to the model developed by them.

The different behavior of potato starch and wheat flour during the penetration test, resulting in distinct shapes of flow charts, a number of peaks, strength values, and linear growths may be due to the separate botanical origin of the materials and, consequently, other properties of the powders. Internal properties, specific for a particular powder, include, among others, parameters describing the morphology of particles, which can have a significant impact on the flow properties of powders. Fitzpatrick et al. [28] analyzed 13 powders with different parameters such as particle size, volume density, particle density and humidity. As a result, the materials were divided into groups and the results showed a significant influence of particle size and moisture on the flow properties of the powders. Molenda et al. [29] showed that potato starch has twice the particle diameter of wheat flour particles, and they also differ in shape. They also observed strong fluctuations in the stress-strain curve and slip-stick in potato starch, which may be the result of a wide range of particle size distributions (eight fractions), while small fluctuations were observed in wheat flour at three particle size fractions. The penetration tests using glass balls of different diameters have also shown that the particle size of the powder can be important for the degree of flowability. The greater the proportion of the fine fraction, the lower the powder flow rate, while an increase in the average particle size led to an increase in flow rate [30]. This means that particle size has an important role to play in material behavior [31]. Additionally, in the case of the studies presented in this article, changes may result from different particle size distributions in the tested materials. Furthermore, on the basis of the conducted experiments and available literature, it can be concluded that tests based on sensor penetration can be used to evaluate bulk flowability and caking degree in small powder samples [12,15,22,30,32].

Moreover, the analyzed materials differ in their chemical composition. In wheat flour, apart from starch, there are additional components such as vitamins, proteins and others [33] which may also affect the analyzed parameters. Additionally, the content of amylose and amylopectin, i.e., the components of starch grains, and their packaging are different in tuber and cereal starches, which may also result in different properties of starch [34].

The ratio of amylose to amylopectin, as well as the previously mentioned particle size and other physical and chemical properties, can also influence the behavior of starch in increased moisture [35]. This is related to the viscosity of starch and the compactness and form of phosphorus. Potato starch has the most phosphorus in the form of monoesters associated with amylopectin, which are responsible for viscosity. Phosphate groups on amylopectin also increase the swelling capacity of potato starch [34]. Thus, it can be concluded that in increased moisture, potato starch becomes more viscous and forms layers more easily. In addition, the viscosity is negatively correlated with the protein content, which means that wheat flour with more protein will have a lower viscosity than potato starch [34].

**Figure 7:**
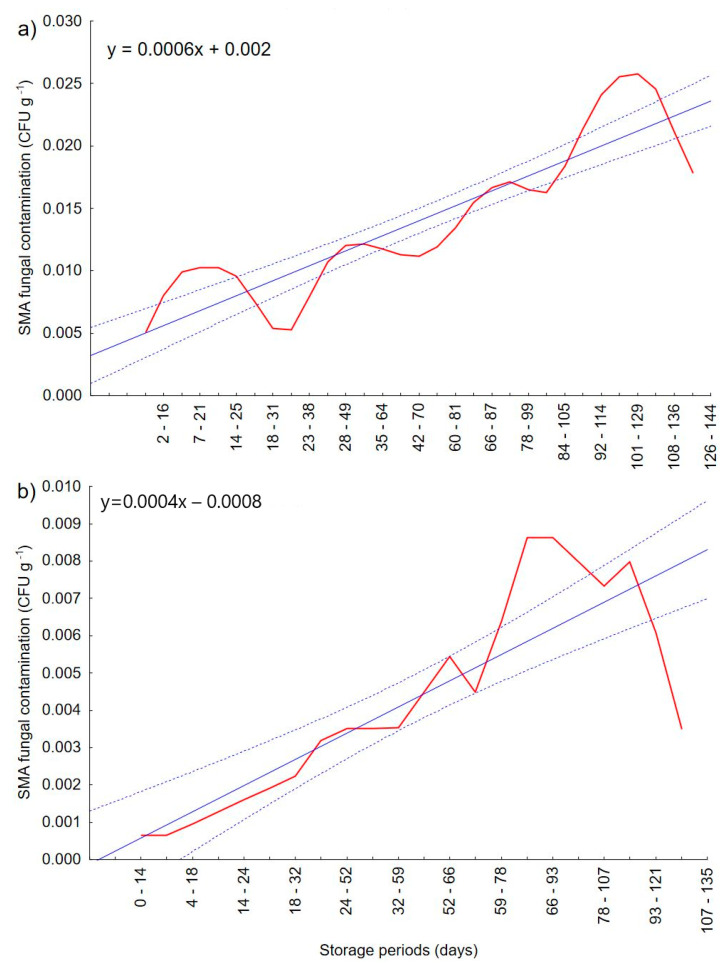
SMA (simple moving average) fungal contamination in (**a**) potato starch (**b**) wheat flour after. The blue continuous line is the trend line, while the dotted line is the 0.95 confidence interval.

The degree of fungal contamination was determined as additional analysis of materials. Figure 7 shows the moving average for fungal contamination with a 0.95 confidence interval. The aim of the applied method was to level out fluctuations, which are very common in such analyses, and additionally to obtain smoother results and easier interpretation. The results show that the amount of mold increases with the storage time. However, this increase is not as high as in the results obtained in the experiment Wajs et al. [22], where one of the storage conditions was high ambient humidity (100% RH). The development of the fungus depends on environmental conditions such as water activity [21], where the most optimal conditions for mold development is an RH value of 85% and more [36]. Therefore, the low CFU values obtained in this experiment may be due to the shoulder of the appropriate conditions for growth. The fact that fungal contamination was very low in the materials tested may suggest that the storage conditions are suitable for these materials and prevent mold growth.

The contamination of wheat flour with fungi was similar to that of potato starch. In wheat flour, the first appearance of fungal contamination at a very low level occurred on the 9th day, slightly earlier than in starch (14th day). This may be due to slightly better conditions for development than in potato starch, due to the compactness of additional ingredients such as proteins, vitamins, etc. [33]. It was observed that over time the number of fungi increased. However, all the values were significantly lower than in potato starch, which is the opposite of the experiment carried out earlier [22], in which higher content of fungi occurred in wheat flour in high humidity conditions. Additionally, no correlation was observed between the strength obtained in the punch test and the number of microorganisms in potato starch and wheat flour. The opposite conclusions were obtained in the publication Wajs et. al. [22], where an increase in the number of molds significantly affected the decrease in agglomerate strength. In the case of this experiment, it is difficult to speak about the effect on caking. It may result from the previously mentioned too small number of fungal impurities. Based on the moving average graphs, it can be concluded that the upward trend in the number of microorganisms may continue and could affect caking over an even longer storage period. For this purpose, further studies are necessary.

## 4. Conclusions

The longer the potato starch was exposed to moisture, the higher the depth of the compacted material, which can be observed in the form of shortened linear growth segments and an increasing number of peaks.

The conditions of 75% humidity and 21 °C can be regarded as the beginning of the caking process. Adsorption of water from the environment contributes to an increase in the concentration of starch and the formation of material layers. As the moisture content of the material increases, the risk of caking in the material and settling on the walls of storage tanks increases.

Different punch test graphs in wheat flour and potato starch suggest that the caking process is different in both powders. The shortening of the linear growths and the zoom of the peaks in potato starch may show a gradual clumping at several points in the sample and then the joining of the resulting thickened layers, while the wheat flour is homogeneous in the entire volume of the sample.

The low strength values of wheat flour suggest that this material has a low affinity for caking under the given conditions.

The correlation of the shortening of the first linear growth section in potato starch, with the development of fungal contamination, may suggest that mold affects caking. However, the amount of mold in both wheat flour and potato starch is very low. To completely exclude or confirm the effect of fungal contamination on the caking phenomenon, an experiment with the above-mentioned powders should be carried out.

The increase of fungal impurities in the potato starch under the conditions of 21 °C 75% RH begins from day 42; therefore, it can be assumed that until this day the material is not microbiologically contaminated. However, this concerns mold and yeast only. After that time, to use the starch, the fungal contamination level of the material should be performed, and other groups of microorganisms should be tested.

## Data Availability

Data sharing not applicable.
